# 

**Published:** 1871

**Authors:** 


					﻿PUBLISHED MONTHLY, AT $3 00 PER ANNUM IN ADVANCE.
ATLANTA
Medical and Surgical Journal.
]•. D Ill. D B Y
W. F. WESTMORELAND, M. D„
Prof, of the Principles and Practice of Surgery in the Atlanta Medical College.
and c
J. G. WESTMORELAND, M. D.,
Prof, of Materia Medica and Therapeutics in Atlanta Medical College.
Pax et Scientia, sed veritas sine timore.
Volume IXB JULY AND AUGUST, 1871. Nos 4 & 5.
ATLANTA GEORGIA.
J'HE JrL'E pEORGlAN J?OOK AND jlOB PRINTING OFFICE
1871.
EACH NUMBER CONTAINS SIXTY-FOUR PAGES.
				

